# Challenges in Treating Low Blood Pressure in Preterm Infants

**DOI:** 10.3390/children2020272

**Published:** 2015-06-15

**Authors:** Eugene M. Dempsey

**Affiliations:** 1 Department of Paediatrics and Child Health, University College Cork, Cork City post code, Ireland; 2 Irish Centre for Fetal and Neonatal Translational Research (INFANT), Cork, Ireland; 3 Department of Paediatrics and Child Health, Cork University Maternity Hospital, Wilton Cork, Ireland; E-Mail: g.dempsey@ucc.ie; Tel.: +353-21-492-0525; Fax: +353-21-492-0770

**Keywords:** Hypotension, blood flow, dopamine, extreme preterm infant, intravetricular haemorrhage

## Abstract

Whilst the prevalence of low blood pressure in preterm infants seems to have fallen over the last number of years, the problem is still frequently encountered in the neonatal intensive care unit and many babies continue to receive intervention. Great variability in practice persists, with a significant number of extremely low gestational age newborns in some institutions receiving some form of intervention, and in other units substantially less. A great degree of this variability relates to the actual criteria used to define hypotension, with some using blood pressure values alone to direct therapy and others using a combination of clinical, biochemical and echocardiography findings. The choice of intervention remains unresolved with the majority of centres continuing to administer volume followed by dopamine as a first line inotrope/vasopressor agent. Despite over 40 years of use there is little evidence that dopamine is of benefit both in the short term and long-term. Long-term follow up is available in only two randomised trials, which included a total of 99 babies. An under recognized problem relates to the administration of inotrope infusions in very preterm infants. There are no pediatric specific inotrope formulations available and so risks of errors in preparation and administration remain. This manuscript outlines these challenges and proposes some potential solutions.

## 1. Introduction

The decision to intervene in preterm infants with low blood pressure remains unresolved and as a result significant variability in practice remains [1,2]. The recent ELGAN study reported significant variation across 14 centres in the United States. In one centre almost all (98%) ELGANS admitted to the intensive care unit received volume, and in the same centre over 64% of *all* ELGANs received inotrope therapy. This compares to less than 30% receiving volume and 6% receiving an inotrope in another centre [3]. Undoubtedly case mix will account for some of this variability, but it is difficult to see how this can account for such wide variation (64% *vs*. 6%). Differing practices must account for a large proportion of this variability. A recent survey highlighted the lack of protocols in many institutions [4]. The most consistent finding of the ELGAN study is that in the majority of circumstances intervention commenced on the first day of life (90%, 89%, 91%, and 89% of infants born at 23 to 24 weeks, 25 weeks, 26 weeks, and 27 weeks of gestation). This is a time period where notable cardiorespiratory changes occur. Treating physiological adaptation may not be the best course of action.

A consistent finding from many surveys of practice is that in the majority of cases intervention is driven by blood pressure values [2,4,5,6,7]. A Canadian survey of over 120 neonatologists identified the BAPM guideline of a mean blood pressure less than gestational age as the commonest criterion used for intervention by over 87% of respondents [7]. Seghal and colleagues in a similar study conducted in Australia and New Zeeland found that a mean blood pressure was the single most important criteria used to determine intervention [5]. The most recent survey, conducted by the HIP consortium, identified that the BAPM rule was used by 75% of respondents to define a low blood pressure that may warrant intervention [4]. This is an important finding and shows that very little has changed in practice over the last 10 years. The HIP survey is important as it was a multinational survey with over 200 neonatologists responding. The fact that blood pressure values are used to guide intervention should not come as a surprise to many. Invasive blood pressure values are, generally speaking, easy to obtain at the bedside. They are objective, provide a continuous bedside value to the clinician, and the effects of intervention can be easily monitored. Low blood pressure values have been associated with adverse long-term neurodevelopmental outcome [8]. However, there are two key concerns that need to be highlighted: (1) The relationship between flow and blood pressure is poorly correlated; and (2) The relationship between low blood pressure and adverse long-term neurodevelopmental outcome remains to be determined.

## 2. Blood Pressure and Flow

“It is a source of regret that measurement of flow is much more difficult than measurement of pressure. This has led to an undue interest in blood pressure measurements.” This statement by Austrian physician Adolf Jarisch Jr, almost 100 years ago, still holds true today for the clinician at the bedside. Historically, for reasons mentioned above, clinicians have continued to be guided by blood pressure values. However, the relationship between flow and pressure is complex, especially when one factors in the dynamic changes that are taking place during the first 24 hours of life. A number of authors have highlighted the poor relationship between flow and blood pressure in preterm infants in the first few days of life [9,10,11,12,13]. There is consistently a poor relationship between measurements of superior vena cava flow and mean arterial blood pressure, and also a poor relationship between right and left ventricular output and mean arterial blood pressure. This should not be surprising as blood flow is dependent on two key factors, blood pressure and vascular resistance (Cardiac output = Blood pressure/systemic vascular resistance). If the mean arterial blood pressure is low but the vascular resistance is low, then flow can be normal or even high. Likewise the mean arterial blood pressure may be normal but if the vascular resistance is high then the blood flow may be suboptimal. Therefore, normal pressure should not assume normal flow. Echocardiography measurements of output can be challenging to obtain especially in small critically ill newborns. These measurements are not continuous, are prone to inter and intra-rater variability [14,15], and require a degree of handling if one is to perform repeated measurements [16]. Other methods of non-invasive assessment of flow are essential to guide therapy and these will be discussed later.

## 3. Low Blood Pressure and Adverse Outcome

Numerous studies have highlighted an adverse relationship between low blood pressure and adverse outcome. We explored this relationship previously and highlighted some of the concerns around this relationship [17]. We identified 18 studies including cohort studies and case controlled studies with small numbers of patients and none of which were powered for long-term neurodevelopmental follow up. The overall assessment of the data was that there is some association between having a lower blood pressure and having a worse outcome but that there were several potential confounding factors that precluded one from drawing any strong inference from this association. The definition of hypotension varied substantially across the studies. An often used definition is a single mean blood pressure value less than 30 mmHg. This is an unsound definition of hypotension and may result in an artifactual association between hypotension and adverse outcome. The more immature babies, at greatest risk for IVH, are much more likely to be hypotensive if one used a cut off of 30 mmHg.

Other authors have also questioned the relationship between low blood pressure and adverse long term outcome. Logan and colleagues evaluated three indicators of hypotension: (1) lowest mean arterial pressure (MAP) in the lowest quartile for gestational age; (2) treatment with a vasopressor; and (3) blood pressure lability, defined as the upper quartile of the difference between each infant’s lowest and highest MAP. They found no association between any of these definitions and adverse outcome defined as white matter abnormality on cranial ultrasound or a diagnosis of CP at 24 months [18]. In a similar study, they found little evidence that the early postnatal hypotension indicators, as defined above, were associated with developmental delay at 24 months corrected gestational age [19]. Van Bel and colleagues found that a MABP less than GA (in weeks) was not associated with lower cerebral oxygenation values (rScO2) or with lower neurodevelopmental outcome scores. Instead, they found that low rScO2 was associated with lower neurodevelopmental outcome scores. This study highlights end organ assessment, namely cerebral oxygenation, as a potential marker of long term neurodisability. The authors concluded by suggesting that preterm neonates could benefit from the inclusion of NIRS in (hypotension) treatment protocols [20].

Others have suggested that it is the treatment that may be a factor in adverse outcome [21,22,23,24]. Fanaroff found an association between treated hypotension and adverse outcome defined as delayed motor development, hearing loss, and death [21]. Limperopoulos found an association between volume expansion on the second day of life and abnormal cranial ultrasound finings [23]. Data from the Canadian Neonatal Network (CNN) showed that infants with hypotension defined as a lowest blood pressure less than their gestational age, or a blood pressure less than the tenth percentile using Watkins’ criteria, were statistically more likely to have severe IVH. However, this was no longer apparent when the use of blood pressure agents were included [24]. A relatively important and interesting finding was that patients who were normotensive yet treated had a worse outcome than infants who were hypotensive and not treated. Rather than assigning blame to inotrope administration, it is feasible that clinicians recognized other important variables that directed treatment and so a more global approach rather than just blood pressure values were included in the decision to treat. However, it is not feasible to answer this question retrospectively, but it does call into question the overall approach to treatment in this population of infants. Future clinical trials, adequately powered to address long-term outcome, are now required.

## 4. Assessment of Circulatory Well-Being

Circulatory well-being is generally assessed clinically at the bedside [13]. However, this assessment can be quite subjective and as a result can lead to significant variation in assessment. De Backer *et al* recently described three clinical windows at the bedside in the overall assessment of circulatory shock, namely the skin, kidney and brain [25]. Clinicians often describe the infant as being pink, well perfused, having adequate urine output and good overall level of activity. We previously described a cohort of infants who had low blood pressure, clinically appeared well and who had a good short term clinical outcome: referred to as permissive hypotension [26]. However, the lack of objective criteria to evaluate circulatory wellbeing persists. A recent European survey highlighted that these clinical parameters were included, but also biochemical parameters such as lactate, pH, base deficit, and haemoglobin values [4]. Each of the parameters in isolation have a low sensitivity for detecting low flow states, but when combined provide a better global assessment of circulatory well-being [13,26,27]. DeBoode provides an excellent overview of noninvasive assessment of circulatory well-being and highlights the importance of global assessment [28]. Other ancillary investigations are now growing in popularity, including the use of functional echocardiography [29,30,31], near infrared spectroscopy [32,33,34] and other methods of noninvasive continuous assessments such as perfusion index measurements [35] and noninvasive cardiac output measurements [36,37,38].

## 5. Prevention

Optimising transition: A great deal of interest and a number of clinical trials have reported the effects of placental transfusion on outcome in preterm infants.

A recent systematic review by Rabe *et al*. [39] found a lower mean blood pressure and a greater need for inotrope support in the group who received immediate cord clamping (ICC) compared with those who had delayed cord clamping (DCC). No measures of cardiac output were evaluated in this review. However Somers, in a nested cohort study of 51 preterm infants, found no differences between groups in heart rate and mean blood pressure, but did find a difference in svc flow measurements over the first four days of life [40]. Oh also found no difference in the hourly mean arterial blood pressure during the first 12 h of life [41]. A recent meta-analysis identified two studies that reported on blood pressure data following umbilical cord milking (UCM) [42]. Systolic, diastolic, and mean blood pressure were all significantly higher in the group who had umbilical cord milking performed. However, there was significant heterogeneity in the studies and as mentioned previously, no consensus on what constitutes UCM. Finer found similar mean blood pressure values between a group who received UCM *versus* ICC [43]. Whilst there was no difference in blood pressure values, there was a significant difference in SVC over the first 24 h. However, this did not translate into a difference in the need for volume, pressor, or hydrocortisone therapy. A recent meta-analysis of UCM found that whilst UCM was associated with some benefits and no adverse effects in the immediate postnatal period in preterm infants, further studies were warranted to assess the effect of UCM on short-term and long-term outcomes [44]. Some recent work involving animals has provided evidence of the importance of appropriate ventilation at the time of DCC. In preterm lambs with delaying cord clamping for 3–4 min until after ventilation is established improves cardiovascular function [45]. However, the results of the Australian Placental Transfusion Study Echo Sub-Study [46] found no difference in the incidence of low blood pressure (gestational age based rule for 15 min) between both groups and also no difference in the use of inotropes between both groups.

Optimising Respiratory Support: A recent review article by Polgaise and colleagues highlights the complex cardiorespiratory changes that occur over the first few minutes of life as newborns adapt to extrauterine existence, [47] and the potential influence of manual ventilation strategies on this adaptive process. This does raise concerns regarding the current sustained inflation strategies being studied. Kluckow previously highlighted the effects of an alteration of PEEP from 5–8 in ventilated preterms and showed a significant reduction in right ventricular output [48]. Ventilation strategies that aim to avoid mechanical ventilation seem to have a lower incidence of low blood pressure and low flow states [49]. De Waal and colleagues describe haemodynamic variables in infants who have had the INSURE procedure and describe an incidence of hypotension of 16% and rates of intervention at 10%. Similar data is presented for The Avoidance of Mechanical ventilation trial [50] where rates of 17%–18% of patients had low blood pressure. Therefore, although the evidence is primarily from cohort studies it would appear that mechanical ventilation can have an adverse effect on cardiovascular status and should be a recognized cause of low blood pressure/low flow in preterm infants. In the Neopain Trial in which almost 900 ventilated preterm infants were randomised to morphine or placebo, hypotension was associated with the use of morphine [51]. Therefore, strategies to minimize mechanical ventilation should form a critical part of the management of any preterm infant with low blood pressure.

## 6. Therapeutic Interventions

Algorithms outlining the treatment of low blood pressure and surveys of practice are characterized by a very similar approach, volume administration initially followed by dopamine. However, there is little evidence to support this approach.

### 6.1. Volume

Volume administration, typically 10 mls/kg of normal saline, is the initial intervention by most clinicians to hypotension in the preterm infant [7]. However, the majority of preterm infants with hypotension in the first few days of life are not hypovolaemic and have normal circulating blood volumes. This approach may be unwarranted and potentially detrimental. The relative lack of response to volume administration is further evidence that hypotensive preterm infants are not hypovolemic. There are a few circumstances where volume support is crucial, such as in sepsis, but the incidence of early onset sepsis is low and accounts for only a small percentage of hypotensive preterm infants in the first few days of life. Volume administration may seem relatively innocuous but increased fluid administration during the first few days of life is associated with adverse outcome including an increased prevalence of bronchopulmonary dysplasia (BPD) and an increase in the incidence of IVH in preterm infants receiving rapid volume expansion [52,53,54]. Adverse neurologic outcome has been documented in VLBW infants who had received colloid boluses. Therefore the routine use of volume in transitional low blood pressure remains a practice without any supporting evidence and judicious use of volume should be the norm [55,56,57].

### 6.2. Catecholamines

One way to assess the efficacy of catecholamines is to assess these agents based on the goals of one’s intervention or so called “goal directed therapy”. For example when a clinician decides to start an inotrope is the aim to increase the blood pressure, or is it to improve end organ blood flow, thus preventing brain injury and improving long-term outcome? These can be thought of as immediate, short term, and long term outcomes of the intervention. The primary aim at present is increasing blood pressure, for all the reasons stated previously. Dopamine is the commonest inotrope used in this population of infants and has been in use in the newborn infant for approximately 40 years. However, there are no studies comparing dopamine *versus* placebo in patients with low blood pressure or low flow states. There are a number of studies of dopamine *versus* placebo but these are in infants who are not hypotensive [58,59,60]. There are now 11 randomised trials of dopamine *versus* another inotrope in patients with hypotension or low flow states; dobutamine, eight trials [61,62,63,64,65,66,67,68], epinephrine two trials [69,70] and most recently vasopressin, in one trial [71]. All of these trials are characterized by variability; in patient populations, in patient numbers, definitions of hypotension, different inclusion criteria and different outcome measures (See Table 1 and Table 2). Dopamine reliably increases blood pressure but seems to do so largely by increasing vascular resistance, which may subsequently lead to a decrease in systemic perfusion [65,72]. The only certainty is that dopamine is more effective than dobutamine at increasing blood pressure and is characterized by less treatment failure, less cumulative dose, and a faster attainment of blood pressure [73]. Despite this it is unclear if it has any other short term benefits and certainly there is no evidence that dopamine has any long-term benefits. It is associated with a reduction in T4 and TSH levels, which although reversible on cessation of therapy, [66] may potentially have adverse long-term effects. Dobutamine does not reliably increase blood pressure but usually is associated with an increase in left ventricular output and SVC flow [65]. There is little long-term data on dobutamine use to make any meaningful comment [74]. Epinephrine is as effective as dopamine at increasing blood pressure and is associated with an improvement in left and right ventricular output [69], but again there is little evidence of any benefit in meaningful short-term outcomes and no evidence of any benefit in the long-term [75].

**Table 1:** Dopamine *versus* Dobutamine in preterm infants with cardiovascular instability.

Author	Number of Patients	Gestation/Wt	Definition
Roze	20	<32 weeks	>30 mmHg
Klarr	63	<34 weeks	>30 mmHg
Greenough	40	<33 weeks	Systolic < 40 mmHg
Hentschel	20	25–36 weeks	Not stated
Chatterjee	20	<32 weeks	Not stated
Ruelas-orozco	63	1.0–1.5 Kg	<30 mmHg
Fillipi	35	<0.75, <1.0, <1.5 Kg	<25, <30, <32 mmHg
Osborn	40	<30 weeks	Low flow

**Table 2:** Cardiac output meaurements in patients treated for cardiovascular instability.

*Study*	*No.*	*Gestation*	*Agents*	*RVO*	*LVO*	*SVC*	*Outcome*
Roze	20	<32 weeks	Dop Dob	n	y	n	Dop: Reduction
Chatterjee	20	<32 weeks	Dop Dob	y	y	n	No change
Phillipos	20	>1750 g	Dop Epi	y	y	n	Dop: Reduction
Osborn	40	<30 weeks	Dop Dob	y	n	y	Dop: Reduction
Lundstrum	36	<33 weeks	Dop Vol	n	y	n	Dop: Increase
Paradisis	90	<30 weeks	Mil Pla	y	n	y	No change

Dop: Dopamine; Dob: Dobutamine; Epi: Epinephrine; Mil: Milrinone; Vol: Volume; Pla: Placebo.

### 6.3. Milrinone

Milrinone, a phosphodiesterase inhibitor is commonly used in pediatric intensive care units, typically following cardiac surgery. It improves contractility and reduces pulmonary vascular resistance. However, due to changes in developmental regulation of PDE subtypes after delivery, milrinone might not be an effective inotrope in the immediate transitional period [76]. There are now a number of case reports and series of its use in term newborns with pulmonary hypertension [77,78,79,80]. Typically milrinone requires a loading dose followed by a maintenance infusion. The loading dose is often associated with a lowering of mean blood pressure and this may be one reason why it has not grown in popularity in preterm infants. There is one randomized trial of its use in preterm infants who are at risk of low flow states in the first few days of life [81]. This two centre study enrolled preterm infants less than 30 weeks gestation within the first six hours of life who were deemed to be at risk of developing a low flow state (all infants less than 28 weeks and additional criteria for those above 28 weeks). No significant difference was observed in SVC flow, right ventricular output, and blood pressure during the first 24 hours of life; or instances of Grades 3 to 4 periventricular/intraventricular hemorrhage or death. Constriction of the ductus was slower in the infants randomized to milrinone. No further randomized trials have been performed using milrinone in the first few days of life. There is a case series describing its use in preterm infants following patent ductus arteriosus ligation and it may be a useful therapy for post ligation cardiovascular instability [82]. There is also a case series addressing it’s potential use in preterm infants with pulmonary hypertension [83].

### 6.4. Corticosteroids

Corticosteroids do increase blood pressure in preterm infants. They are now more commonly prescribed for hypotension than for bronchopulmonary dysplasia in premature newborn infants [84]. There are a number of prospective randomised controlled trials addressing corticosteroid use for both the prevention (two trials) [85,86] and treatment (seven trials) of hypotension [87,88,89,90,91,92,93]. Prophylactic use of corticosteroids should be avoided. There are many uncertainties around corticosteroid use in preterm infants with hypotension. Clearly hydrocortisone has a limited role to play. However, the optimal dose and frequency of administration remains unclear. The current trials vary substantially in the dosing regimes with a fifteen fold difference in dose between some studies. Also, concerns around adverse short term and long term side effects are unresolved. If hydrocortisone is to be administered in the first few days of life then the lowest effective dose should be used, and a starting dose of hydrocortisone 1 mg/kg seems reasonable whilst further doses should be determined by the response. This is more a pragmatic approach as opposed to an evidence based approach. A recent randomized trial assessing the feasibility of conducting an RCT in hypotensive preterm infants had a very low overall recruitment rate, which may reflect clinicians concerns in relation to hydrocortisone use with indomethacin administration [94].

## 7. Administration Challenges

There are a number of important factors that need to be considered when delivering an inotrope infusion in very preterm infants, factors that are not as relevant in the paediatric/adult population. The first relates to the drug formulation; firstly there are no neonatal specific formulations, a finding that is consistent with the overall lack of newborn specific formulations [95], and secondly concerns related to drug formulations related to excipient use and their potential adverse effects [96,97].

Practical administration challenges relate to issues such as the total daily fluid volume administered in the preterm infant; it may be as low as 30–40 mls in total for a preterm infant weighing 500 grams. As a result every effort is made to ensure optimal administration of parenteral nutrition, and consequently additional infusions volumes need to be low, resulting in low infusion rates e.g., as low as 0.1 mL/h. This brings challenges in relation to optimal drug delivery time especially when taking into consideration dead space issues of the vascular catheter and the additional tubing that may be in place. There can be long lag times before the agent actually reaches the babies bloodstream, an infusion running at 0.1 mL/h may need 90 min to cover a dead space of 0.15 mls (start-up times). This phenomena is often under recognized and can result in both a delay in delivery and unpredictable effects.

There are a number of other important factors that need to be considered that can influence the delivery of an agent. It is best to think of these from the syringe driver through to the vascular access device. These include the vertical displacement of the syringe pump, the syringe itself, the tubing, the vascular access device being used and finally the concomitant infusions that may be running. Upward displacement results in a reduction in the internal pressure of the tubing and ultimately an increase in the flow rate, resulting in a bolus delivery. The lower the infusion rate, the greater the bolus delivery. Conversely, a downward displacement will result in an increase in the IP and a reduction in the flow rate. A number of studies on animals have highlighted these findings [98,99,100,101].

Studies on syringe size and design have shown that the time to reach steady-state flow, time to occlusion alarm, and the time without flow, increase with syringe size and lower preprogrammed flow rates. Increased syringe size will result in increased bolus volume caused by vertical displacement. The tubing compliance will also influence the time to occlusion alarm. A low compliance tubing will result in an earlier alarm than a high compliance tubing system. It is essential that clinicians are familiar with dead space volume issues of their own vascular device; either the umbilical venous catheter or PICC line. As is often the case in newborn care vascular access is limited and multiple infusions are often run in a single catheter. The carrier flow rate is important as low total flow results in a slower time to reach a steady state compared to high flow rates. Perhaps the most important aspect is that clinicians and healthcare professionals become educated in the concept of flow variability and the factors that may influence this variability. Efforts should be made to ensure low occlusion pressures are used, highest possible/practical flow rates are utilized, low compliant tubing and minimize vertical displacement of the syringe pump relative to the patient [101].

## 8. Prospective Trials

In the last few years a number of studies in the area of cardiovascular support in preterm infants have commenced. Two of these studies have ceased recruiting, namely the HIPHOP trial from Italy and the NICHD hypotension trial [94]. Both studies ended prematurely because of significant issues around patient recruitment. The NICHD trial enrolled in total 16 patients from an initial total of 120 eligible patients. A number of factors contributed to this low overall rate of enrolment, including consent issues and physician willingness to enroll. The factorial design meant that half of those enrolled would receive hydrocortisone, either alone or with dopamine. Many centres were using prophylactic indomethacin and hence the concern in relation to intestinal perforation meant physicians were less likely to approach and consent parents [94,102]. The HIPHOP trial (Eudract 2009 – 016653 – 17) has yet to report its results but again ceased early because of enrolment issues (personal correspondence from the lead clinician).

Whilst I have highlighted a number of the challenges in this area, it is encouraging to see a number of clinical trials ongoing. The European Commission has funded two of these studies in an attempt to determine the efficacy of dopamine and dobutamine in the management of cardiovascular compromise. The HIP trial (http://www.hip-trial.com) is now enrolling and is attempting to determine whether a standard approach to management of hypotension with volume and dopamine, *versus* a more observational approach with placebo (permissive approach), will result in improved short and long-term outcomes for preterm infants less than 28 weeks [103]. The Neocirculation group (http://neocirculation.eu/) will determine if dobutamine therapy results in an improvement in outcome in preterm infants with low flow as evidenced by low superior vena cava flow determined by echo. The TOHOP study is currently enrolling patients with low blood pressure and is attempting to evaluate the role of near infrared spectroscopy as an adjunct in the management of low blood pressure, focusing on an objective assessment of end organ blood flow. These studies will hopefully provide the clinician with evidence to direct therapy in preterm infants in the first few days of life.

A recent randomized trial utilising near infrared spectroscopy with an associated treatment algorithm in preterm infants less than 28 weeks is a novel approach to managing preterm infants in the first few days of life. Patients were randomized to either visible monitoring with the treatment algorithm, or standard care. If cerebral saturations were above 85% or less than 55% in the monitored group then a list of options were available to the clinician. This is a paradigm shift in newborn care, focusing on the end organ. The primary outcome was a reduction in the time spent “outside the normal range”. The monitored group spent a significantly shorter percentage of hours outside this range. Whilst there were no statistically significant differences in secondary endpoints, fewer patients died in the monitored group compared to the non-monitored (16% *versus* 25%), prompting the need for a much larger study.

## 9. Conclusions

Many preterm infants continue to receive treatment for low blood pressure. The main treatment criterion used is having a mean blood pressure less than gestational age, and the main treatment approach is volume administration followed by dopamine. The evidence is lacking for both the definition and this current management approach. This review article has highlighted that a number of key issues remain unresolved. What should direct intervention? Who should be treated? Does treatment improve long-term outcome? It is encouraging to see a number of clinical trials currently underway in an attempt to finally address these important issues. Whilst we will have to wait for a number of years until these trials are completed, the judicious use of inotropes in this population is warranted.
